# The effect of automated audit and feedback on data completeness in the electronic health record of the general physician: protocol for a cluster randomized controlled trial

**DOI:** 10.1186/s13063-021-05259-9

**Published:** 2021-05-04

**Authors:** Steve Van den Bulck, Tine De Burghgraeve, Willem Raat, Pavlos Mamouris, Patrick Coursier, Patrik Vankrunkelsven, Geert Goderis, Rosella Hermens, Gijs Van Pottelbergh, Bert Vaes

**Affiliations:** 1 Academic Center for General Practice, KU Leuven, Kapucijnenvoer 33 blok J, 3000 Leuven, Belgium; 2 IQ Healthcare, Radboud University Medical Center Nijmegen, PO Box 9101, 6500 HB Nijmegen, The Netherlands

## Abstract

**Background:**

The electronic health record (EHR) of the general physician (GP) is an important tool that can be used to assess and improve the quality of healthcare. However, there are some problems when (re) using the data gathered in the EHR for quality assessments. One problem is the lack of data completeness in the EHR. Audit and feedback (A&F) is a well-known quality intervention that can improve the quality of healthcare. We hypothesize that an automated A&F intervention can be adapted to improve the data completeness of the EHR of the GP, more specifically, the number of correctly registered diagnoses of type 2 diabetes and chronic kidney disease.

**Methods:**

This study is a pragmatic cluster randomized controlled trial with an intervention at the level of GP practice. The intervention consists of an audit and extended electronically delivered feedback with multiple components that will be delivered 4 times electronically to general practices over 12 months. The data will be analyzed on an aggregated level (per GP practice). The primary outcome is the percentage of correctly registered diagnoses of type 2 diabetes. The key secondary outcome is the registration of chronic kidney disease. Exploratory secondary outcomes are the registration of heart failure, biometric data and lifestyle habits, and the evolution of 4 different EHR-extractable quality indicators.

**Discussion:**

This cluster randomized controlled trial intends to primarily improve the registration of type 2 diabetes in the EHR of the GP and to secondarily improve the registration of chronic kidney disease. In addition, the registration of heart failure, lifestyle parameters, and biometric data in the EHR of the GP are explored together with 4 EHR-extractable quality indicators. By doing so, this study aims to improve the data completeness of the EHR, paving the way for future quality assessments.

**Trial registration:**

ClinicalTrials.gov NCT04388228. Registered on May 14, 2020.

**Supplementary Information:**

The online version contains supplementary material available at 10.1186/s13063-021-05259-9.

## Background

The electronic health record (EHR) is an important instrument that, when properly implemented, can be used as a tool to improve the quality of healthcare [1]. However, many challenges need to be overcome to use the vast amount of EHR-stored data for quality assessment [2, 3]. One of these challenges is the lack of data completeness of the EHR and, in particular, the varying quality of the correct and complete registration of diagnoses [4–6]. After all, when the quality of care for certain diseases needs to be evaluated, the number of registered diagnoses of these diseases in the problem list of the EHR needs to be as accurate as possible to have reliable results in our quality measurement. In the past, data quality feedback tools and frameworks have been developed to address the problem of the lack of data completeness [7, 8].

Chronic diseases such as diabetes mellitus type 2 (DM 2) and chronic kidney disease (CKD) have a high prevalence, and the quality of primary care for these conditions needs improvement [9, 10]. For example, CKD can be identified in the EHR with the help of the electronic CKD phenotype, which has proven to be an accurate method to detect patients likely to have CKD based on data stored in the EHR [11]. However, there is still the need for effective organizational and technical strategies to achieve data completeness [12]. The electronic CKD phenotype is thus an important first step, but additional pragmatic trials to explore implementation strategies incorporating this electronic CKD phenotype are necessary [13]. An implementation strategy that can be useful for this purpose in primary care could be audit and feedback (A&F).

Audit and feedback is a well-known quality intervention that, according to the last Cochrane review, leads to “small but potentially important improvements in professional practice” [14]. However, the important features of A&F and their influence on the effect of an A&F intervention are still the subject of debate [15]. Previous work identified some testable and theory-informed hypotheses for designing an A&F intervention, and suggestions to improve the interventions’ effectiveness are available in the literature [16, 17]. Some features of the feedback are known to be effective, for example, the frequency of the feedback provision (more than once), but other features, such as the use of benchmarks as a comparison, the evidence-based quality of the feedback, and a low cognitive load of the feedback, need further investigation [14, 16–19]. Therefore, the aim of this study is to investigate whether an automated A&F intervention, using different features of feedback, can be effective in improving the data completeness of the EHR in primary care.

## Methods

### Study design and objectives

#### Study design

**Fig. 1:**
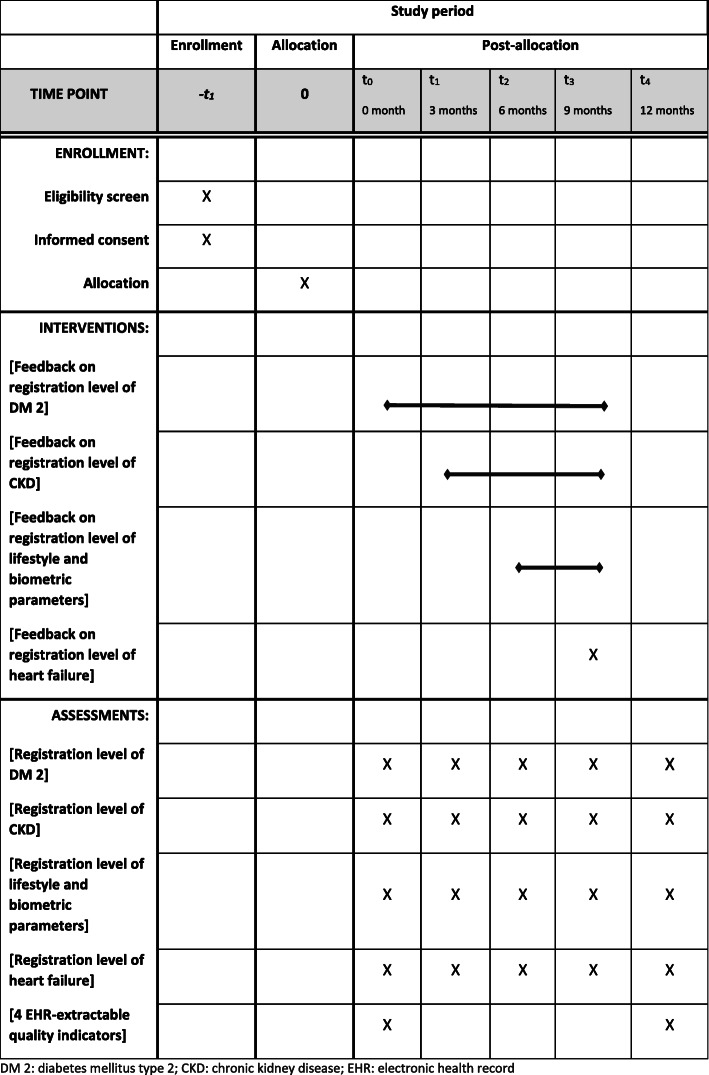
Time schedule. DM 2, diabetes mellitus type 2; CKD, chronic kidney disease; EHR, electronic health record

The study is a pragmatic cluster randomized controlled trial (CRCT) with a superiority framework and an intervention at the level of the general physician (GP) practice. The protocol is reported according to the Standard Protocol Items: Recommendations for Interventional Trials (SPIRIT) checklist [20] (see additional file 1 and Fig. 1).

#### Primary objective

The primary objective of this study is to improve the quality of registration of DM 2 in GP EHR.

#### Key secondary objective

- Improve the registration of CKD

#### Exploratory secondary objectives

- Evaluate the registration of heart failure, biometric data, and lifestyle habits (smoking, exercise, weight, height, alcohol use)
- Two different EHR-extractable quality indicators for DM 2 and two different EHR-extractable quality indicators for CKD will be evaluated to investigate whether an improved registration of these two diseases has an impact on EHR-extractable quality indicators.

### Study population, eligibility criteria, and data storage

This trial has GP practices as the level of allocation. All GPs in the Intego network will be asked to participate in this trial. For GPs working in a group practice, the whole group will be asked to collaborate. GP practices are excluded when participation is not unanimous. Intego is a Belgian general practice-based morbidity registration network at the Center of General Practice of the University of Leuven [21]. Intego collects data on health parameters, incidence and prevalence rates, laboratory results, and prescribed drugs for all relevant subgroups. As of 2020, 395 GPs of 106 practices spread throughout Flanders, Belgium, collaborated in Intego. In order to ensure enough GP practices take part in the trial, all the GP practices will be contacted and/or visited individually and asked to take part in this trial. An Intego researcher will explain the study and request informed consent. The researcher will also emphasize that the provision of the feedback is an incentive on its own. Therefore both groups will receive feedback although the feedback in the control group will be basic. In addition, the Intego researcher will also inform the participants that the intervention will not be intrusive since it will be integrated (automated) in the normal workflow of the GP (to optimize the use of the EHR-system).

The inclusion criteria for GP practices participating in this trial are the same criteria as for inclusion in the Intego network, which are explained in more detail elsewhere [21, 22]. Only the aggregated data of patients 40 years or older who had at least one consultation the past year will be analyzed for both primary and secondary outcomes.

The data will be hosted on the Healthdata.be platform, which is described in more detail elsewhere [22].

### Intervention

The intervention consists of an extended electronically delivered feedback report with multiple components that will be delivered 4 times electronically into GP practices over 12 months. The extended feedback report is generated based on an automated audit that is built into the EHR of the GP. This audit will be executed on the Intego database in an automated fashion 4 times and is available in both the control and intervention groups. The feedback report for both trial arms will be provided on the independent Healtstat.be platform. The intervention group will also receive the extended feedback report through a push system into their e-health inbox to minimize the effort to consult it. The aim of the push system is to involve participating GPs and to actively direct their attention to the task at hand, namely, the registration of diagnosis and parameters in the EHR.

The extended feedback report will be delivered in the form of action plans and goals consisting of multiple components:

- Benchmarking of the results of the audit with peers on disease-specific laboratory results or medication prescriptions. The results of the audit will be compared with the mean of the 10% best performers according to the Achievable Benchmarks of Care (ABC) method [23].
- A low cognitive load of the feedback, such that the results will be presented with the help of graphs and without too many in-depth elements.
- Guidance on which codes to use for which cases or where to put the information in a structured way.
- Links to disease-specific guidelines.
- A push system to minimize the effort the GP must make to consult the feedback.

The aim of the automated audit is to identify the unregistered population, and this built-in audit is available in both study arms. The aim of the extended feedback report is to improve the registration level in the EHR and thus improve the data completeness of the EHR.

The control group will also have access to the automated audit since it is built in the EHR and will receive basic feedback on the level of registration in their EHR. The feedback will only be available on HealtStat.be through a pull system, so GPs will need to actively check Healtstat.be. For a detailed comparison of control versus intervention, please see Table 1.

**Table 1:** Intego feedback trial: control versus intervention group

	Control group	Intervention group
Four different feedback moments and reports over 12 months on the following: - The registration level of DM 2 - The registration level of CKD - The registration level of lifestyle habits: the number of patients with lifestyle factors registered in the EHR; lifestyle factors = smoking, alcohol use, exercise, weight, height - The registration level of heart failure	• Diagnostic built-in audit on registration of (parameters of) DM 2, CKD, heart failure and lifestyle habits available in the EHR CareConnect®	
	• Basic feedback available at Healthstat.be • Feedback = percentages per registered diagnosis/parameter per GP practice in text • Pull system where the GP needs to actively check for feedback by logging in on the website of Healthstat.be	• Extended feedback in the form of action plans and goals consisting of the following: o Percentages per registered diagnoses/parameters per GP practice, presented in graphs with low cognitive load o Benchmarking with peers on disease-specific lab results and/or with the percentage of patients who receive disease-specific medication (ABC method) o Guidance on which codes to use for which cases or where to put the information in a structured way o Links to disease-specific guidelines • Available at Healthstat.be • Push system to eHealth inbox to minimize the effort necessary to consult it

*DM 2* diabetes mellitus type 2, *CKD* chronic kidney disease, *EHR* electronic health record, *GP* general physician, *ABC* Achievable Benchmarks of Care method

### Allocation concealment and random sequence generation

The list of participating practices will be known at the start of the trial. The allocation sequence will be performed by a statistician (independent from the research group) and it will be generated using covariate-based constrained randomization [24]. More specifically, out of a list of all acceptable allocations one allocation scheme will be drawn at random. To balance the mean size of the practice between both groups, a list of all possible allocations (restricted to those allocations with equal number of practices in each group) with a maximal difference of 5% in mean number of patients per practice and a maximal difference of 5% in total number of patients will be created and one allocation will be drawn at random from this list [24]. The covariate-based constrained randomization procedure will be implemented using a SAS-macro [25]. If the trial would start before all practices are known, practices with “late entry” will be allocated using minimization [26], categorizing the number of patients per practice into 2 or 3 categories.

The control versus intervention group will be allocated in a 1:1 manner. GPs are not blinded to the intervention. The research facility will be blinded to the allocations until all data are collected.

### Outcomes

Our primary outcome is the registration level of DM 2. This registration level will be expressed as the percentage of registered diagnoses of DM 2 in the EHR. The numerator will be the number of registered diagnoses of DM 2 in the EHR, and the denominator will be the number of patients with a prescription of diabetes medication (ATC classification A10) or with elevated levels of HbA1c (at least once > 53 mmol/mol) or with abnormal fasting glycemia (2 independent measurements > 126 mg/dl). For the primary outcome, we hypothesize that the intervention can achieve an improvement of 10% points (from 75% to 85%) in registration level within 12 months.

As a key secondary outcome, we will focus on the registration of CKD. The registration of CKD will be expressed as the percentage of registered CKD diagnoses in the EHR, with the numerator the number of registered diagnoses of CKD and the denominator the number of patients with a decreased (≤ 45 mL/min/1.73 m^2^) estimated glomerular filtration rate (eGFR). For the registration of CKD, we hypothesize the intervention can achieve an improvement of 20% points (from 50% to 70%) in registration level because preliminary analyses show the quality of registration of CKD is lower than for the primary outcome.

As exploratory secondary outcomes, we will evaluate the registration of heart failure, lifestyle factors (such as smoking and alcohol use), and biometric data (such as weight and length), and explore two EHR-extractable quality indicators for CKD and two for DM 2. The registration of heart failure will be expressed as the percentage of heart failure patients, with the numerator the number of registered heart failure diagnoses and the denominator the total active patient population older than 40. Lifestyle factors and biometric data will be expressed as the percentage of patients who have registered lifestyle factors or biometric data in their EHR.

The EHR-extractable quality indicators we will explore are as follows [27, 28]:

- Percentage of patients with CKD, in whom the GFR, albuminuria, and total protein is determined at least once a year
- Percentage of patients with CKD who are vaccinated with a pneumococcal vaccine
- Percentage of patients with DM 2 and an eGFR of < 30 mL/min/1.73 m^2^ who no longer receive metformin.
- Percentage of patients with DM 2 whose HbA1c level is measured at least once every 6 months

### Ethical approval

The Intego procedures were approved by the KU Leuven Ethics Committee (nr. ML1723) and by the National Privacy Commission’s Sectoral Committee (decision nr. 13.026 of March 19, 2013). The procedures to collect data by Healthdata.be were approved by the Belgian Privacy Commission on April 17, 2018.

The protocol for this CRCT was approved by the Ethical Commission Research UZ/KU Leuven with number S62753 (May 13, 2019) and number S62753/0001 (March 20, 2020).

#### Trial registration

Registered on May 14, 2020, on ClinicalTrials.gov (number NCT04388228) https://clinicaltrials.gov/ct2/show/NCT04388228.

#### Informed consent

A researcher of the Intego network will be obtaining signed and written informed consent from each collaborating GP practice, and we will ensure that each GP practice is given a full explanation of the protocol in an information letter.

### Data collection

The data collection takes 12 months (see Fig. 1).

The data in the EHR of the Intego practices will be collected by HealthData through eHealth. This is part of the basic Intego project. This collection will be a snapshot of the EHR, which is used daily by GPs during consultation with patients. The data will be hosted on the Healthdata.be platform, which is described in more detail elsewhere [22].

Based on this data collection, the GPs will receive an A&F intervention with feedback reports on 4 different time points (every 3 months). At these time points, we will remind the GPs to work on their registration level of specific parameters or coded diagnoses in the EHR.

Every week, we will collect the data of the same practices, which is part of the basic Intego project. Then, for this A&F project, we will evaluate every 3 months whether the extended feedback led to a significant improvement in the registration level of the intervention group compared with the control group. We will analyze only the data of the practices that signed an informed consent form.

### Power calculation

To measure an improvement in our primary and key secondary outcome, these are the percentage of registered DM 2 and CKD diagnoses in the EHR, a power calculation was performed.

To measure an improvement of 10% points (from 75% to 85%) in our primary outcome with 80% power, an alpha of 0.05, a variance inflation factor (VIF) of 21.12 (based on an intra-class correlation (ICC) of 0.103), we need 56 practices to be included in this CRCT, or 28 practices in each group (Table 2). To measure an improvement of 20% points (from 50% to 70%) in our key secondary outcome with 80% power, an alpha of 0.05, a VIF of 9.1, and an ICC of 0.32, we need 68 practices to be included in this CRCT, or 34 practices in each group (Table 2). To have enough statistical power to measure an improvement in both outcomes, 68 practices will be recruited. The VIF and ICC were calculated based on historical data from the Intego database and were used to correct for clustering.

**Table 2:** Power calculation

	Assumed % correct diagnoses						Required number		
	C	I	Alpha	N0 (total)	ICC	VIF	Mean per practice	N patients	N practices
**Primary**									
Diabetes	0.75	0.85	0.05	500	0.103	21.12	192	10,560	56
**Key secondary**									
CKD	0.50	0.70	0.05	186	0.32	9.1	25	1693	68

Required total number of practices to have at least 80% power for the comparison of two proportions based on a logistic regression model correcting for the clustering of patients within GP practice. ICC and resulting VIF were obtained from historical data. The alpha level has been set at 5% for the primary endpoint and also at 0.05 for the key secondary endpoint (following a hierarchical closed testing procedure)

*C* control group, *I* intervention, *Alpha* alpha-level, *N0* total number of required diagnoses (patients) based on a *χ*^2^-test (hence, ignoring clustering within GP practices), *ICC* intra-class correlation, *VIF* variance inflation factor (taking into account differences in cluster size), *Cluster size* number of diagnoses per practice, *Mean per practice* mean denominator per practice, *N patients* total number of required diagnoses taking into account the clustering, *N practices* required total number of practices. The number of practices is rounded upwards such that an equal number of practices can be allocated to the control and the intervention group

A power calculation for our exploratory secondary outcomes was not performed.

### Analysis

SAS software (SAS Institute Inc., Cary, NC, USA) and R (R Foundation for Statistical Computing, Vienna, Austria) will be used for analysis and for the graphs of the feedback. For this study, data will only be used on an aggregated level (per GP practice). Analysis will be performed using the numerator and denominator of each outcome per GP practice. The results will be reported according to the Consolidated Standards of Reporting Trials (CONSORT) [29]. The statistical model will not be adjusted by for any covariates.

To evaluate and explore the effect of the intervention on the primary and secondary outcome measures, a logistic generalized estimating equations (GEE) model will be used. This model is chosen because it can investigate the average response of an intervention on a population level. The effect of the intervention will be presented as the difference in proportions together with its 95% confidence interval. The GEE model will also be used to estimate the proportion of correctly registered diagnoses/parameters in the 2 allocated groups with their 95% confidence intervals.

## Discussion

### Overview

This cluster randomized controlled trial investigates the effect of an automated A&F intervention to primarily improve the level of registration of DM 2 and to secondarily improve the level of registration of CKD in the EHR of the GP. Furthermore, the registration of heart failure, lifestyle parameters, and biometric data in the EHR of the GP are explored together with 4 EHR-extractable quality indicators. In this way, this study aims to improve the data completeness of the EHR, paving the way for future quality assessments.

By implementing an automated A&F intervention with the help of the Intego database, this CRCT can form a basis for investigating future A&F interventions, which is particularly important for chronic diseases. Chronic diseases such as DM 2 and CKD have a high prevalence, and the quality of primary care for these conditions needs improvement [9, 10]. We already developed a set of EHR-extractable quality indicators for DM 2, CKD, and heart failure, which can be used in future A&F interventions [27, 28, 30]. In addition, large data repositories in primary care, which collect routine primary healthcare data anonymized at the source, can be used to address many research questions of interest [31]. Examples of these are the Intego database we are using, the Dutch NIVEL, and the British Royal College of General Practitioners’ Research and Surveillance Centre [21, 32, 33]. Using a large database consisting of all EHR files of the Intego practices creates an opportunity to power future studies that can measure small differences in effect between groups. Because of this, it will be possible to compare different A&F features on, for example, the content and delivery of the feedback. After all, recent research indicates the need to compare different A&F interventions with each other instead of comparing A&F interventions with control conditions [34].

The feedback features that are implemented in this CRCT are the use of benchmarks, the frequency, the evidence-based aspect, and a low cognitive load of the feedback. These features are only a few of the 313 theory-informed hypotheses that were suggested as potentially important for improving the effectiveness of A&F interventions [16]. However, in the case of primary care, there is evidence that GPs prefer brief feedback interventions and reports with comparisons and best practice guidelines [35]. With this in mind, the aforementioned features concerning the content and delivery of the feedback are chosen for this trial and combined into an action plan. Furthermore, the use of benchmarks is, according to the recent Clinical Performance Feedback Intervention Theory, useful for improving feedback because it will motivate and compare feedback recipients [36]. Because the Intego database gathers data from approximately 400 GPs, it will be possible to benchmark their results with each other and more specifically with the mean of the top 10% performers according to the Achievable Benchmarks of Care method [23]. Finally, the feedback will be given on four different occasions, since the frequency of the feedback delivery (more than once) is already known to be an important factor [14].

### Strengths and limitations

One of the strengths of this study is the underlying generic approach we are implementing for improving the registration level of two chronic diseases (DM 2 and CKD) in the EHR of the GP. This generic approach is an automated A&F intervention that can be adapted for other diseases and with other features of feedback, so that facilitating factors of future interventions can be evaluated and compared. This thus opens up the possibility to step away from two-arm trials (control versus intervention) in favor of head-to-head trials, as suggested by other authors [34]. Implementing future interventions in this manner and using the Intego database as a large sample size, together with our generic approach, offers the opportunity to power studies that can measure small differences in the effectiveness of certain facilitating factors or features of A&F for improving the quality of primary care [34].

Our study also has some limitations. Since we are implementing an automated and EHR-based intervention, potential sources of bias when (re) using data gathered in the EHR need to be considered [31]. However, the generic aspect of our intervention can potentially assist in resolving some of those problems, such as the quality of the primary care EHR data. Another limitation is that if we want to apply automated quality assessment in the form of A&F for specific diseases, quality indicators that are extractable from the EHR of the GP for these diseases need to be available.

## Trial status

The recruitment for the trial will start in January 2021 and is expected to be completed in June 2021. This protocol is version 4.0, dated June 7, 2020.

### Trial funding

The Intego project is funded by the Flemish Agency “Zorg en Gezondheid.” This trial did not receive individual funding. There was no funding for the design of the study and for the collection, analysis, and interpretation of the data. There was also no funding for writing the manuscript.

## Conclusion

This CRCT investigates the effectiveness of an automated A&F intervention in primary care to improve the data completeness of the EHR. Different features concerning the content and delivery of the feedback will be implemented and evaluated.

## Supplementary Information

**Additional file 1.** SPIRIT checklist.

**Additional file 2.** Ethical approval document.

**Additional file 3.** Informed consent (Dutch).

## Data Availability

Data will be available on reasonable request from the corresponding author.

## Abbreviations

EHR: Electronic health record
DM 2: Diabetes mellitus type 2
CKD: Chronic kidney disease
A&F: Audit and feedback
GP: General physician
SPIRIT: Standard Protocol Items: Recommendations for Interventional Trials
CRCT: Cluster randomized controlled trial
ABC: Achievable Benchmarks of Care
eGFR: Estimated glomerular filtration rate
ATC: Anatomical therapeutic chemical code
VIF: Variance inflation factor
ICC: Intraclass correlation
CONSORT: Consolidated Standards of Reporting Trials
GEE model (logistic): Generalized estimating equations model
