# Supplementary material for: The effect of automated audit and feedback on data completeness in the electronic health record of the general physician: protocol for a cluster randomized controlled trial
Source: Trials. 2021 May 4;22:325. doi: 10.1186/s13063-021-05259-9 (PMC8097814; doi:10.1186/s13063-021-05259-9)
Supplement: Supplementary file 2 — Additional file 2. Ethical approval document. [file 13063_2021_5259_MOESM2_ESM.pdf]

Leuven, 20 March 2020

Ethics Committee
Research UZ/KU Leuven
Herestraat 49
B 3000 Leuven (Belgium)

Email : ec@uzleuven.be

Mevr. Tine De Burghgraeve

Our reference:	EudraCT-nr:	Belg. Regnr:
S62753		B322201940240

Audit and feedback for general practitioners in the Intego network

Modification/additional study documents

AMEND-Id: 0001

Dear colleague

The Ethics Committee Research (EC Research) of University Hospitals Leuven (UZ Leuven) has initially given a positive advice for the above mentioned protocol on 4 July 2019.

Documents/answers submitted on 21 February 2020 have been taken into account in the evaluation of the modification.

A favourable advice for this modification was given on 20 March 2020.

The favourable advice concerns:

Protocol
Version 4 dd 20/02/2020

In case the amended protocol contains important information for the participant, this information should be mentioned in an ICF addendum or adapted Informed Consent Form (ICF). These documents are to be submitted (within a maximum timeline of 6 weeks) to the ethics committee in a subsequent substantial amendment.

The following documents were submitted for notification:

Not applicable

EC Research confirms working in accordance with the ICH-GCP principles (International Conference on Harmonization Guidelines on Good Clinical Practice), the latest version of the Declaration of Helsinki, the Oviedo Convention on Human Rights and Biomedicine and applicable laws and regulations.

EC Research confirms that - in case of conflict of interest - involved members do not take part in the vote concerning the study.

List of members: see appendix.

Points of concern: (if applicable)

The conformity of translated documents compared to the Dutch documents, is the responsibility of the sponsor.

In case of modifications to protocol and/or clinical trial agreement for UZ Leuven, they must also be submitted to the Clinical Trial Center (CTC) of UZ Leuven.

We would like to draw your attention to the fact that EC Research expects her initial comments to be taken into account ab initio at the next submission by the same sponsor.

Studies with investigational medicinal products and certain studies with "medical devices" should be submitted by the client (PI or sponsor) to the FAMHP (Federal Agency for Medicines and Health Products).
Studies with investigational medicinal products are only allowed to be conducted, provided that the minister (FAMHP) does not state objections within legal deadlines as described in art. 13 of the Belgian law of 7/5/2004 concerning experiments on the human person.
Certain studies using medical devices are also covered by legal deadlines (KB of 17/3/2009). Please consult the FAMHP website for more information: www.fagg-afmps.be.

Research on embryos in vitro is covered by the law of May 11, 2003. Before the research project can start, such research also requires a positive advice of the Federal Committee for medical and scientific research on embryos in vitro.

Please take into account the regulations of the hospital concerning tissue management and the regulations of the law of December 19, 2008.

This favourable advice of EC Research does not imply that she will assume responsibility for the planned study. You will remain responsible for the study. In addition, you, as involved principal investigator, should ensure that your opinion as an involved researcher is reproduced in publications, reports for the government, etc. which are the result of this study. You are reminded that concerning clinical studies, any observed serious event needs to be reported immediately to the sponsor and the ethics committee, even if the causal relationship with the study is unclear.

We request you to inform us if the study will not be initiated.

*Finally, we request you to report the termination (early or planned) of the study within the legal deadlines and provide the **Clinical Study Report** (CSR) to EC Research.*

In case of a clinical trial (EudraCT), please be informed that the results must be published in the European Clinical Trial Register. The report of these results can be sent to the EC Research as the CSR.

Yours sincerely,

Prof. dr. Minne Casteels
Chair
Ethics Committee Research UZ Leuven

Cc:
FAMHP (Federal Agency for Medicines and Health Products)

CTC (Clinical Trial Center UZ Leuven)

Participating centres

Local Committee	*Principal investigator*	*Date advice*

List of members EC Research UZ/KU Leuven on 20 March 2020:

Chair	prof. dr. Maria-Reinhilde Casteels	Clinical Pharmacology
Vice chair	prof. dr. Dominique Bullens	Paediatrics
	De heer Jean-Jacques Derèze	Medical Legislation
	De heer Mathijs Swaak	Healthy volunteer repres.
	Mevr. Eva Puttevils	Nurse
	Mevr. Katelijne Van Overwalle	Pt representative (alternate)
	Mevr. Katrin Roggeman	Patient representative
	Mevr. Lia De Wilde	Pt representative (alternate)
	Mevr. Liliane Vandergeeten	Pt representative (alternate)
	Mevr. Marilien Vandeputte	Nurse
	Mevr. Teresia De Fraye	Pt representative (alternate)
	Mevr. Veerle Vanparys	Pharmacist (alternate)
	apr. J.R. Thomas	Clinical Pharmacology
	apr. Stefanie Goris	Pharmacist (alternate)
	dr. Kristel Van Landuyt	Reumatology
	dr. Lut De Groote	General Practitioner
	dr. Marleen Renard	Paediatrics
	prof. André Loeckx	Pt representative (alternate)
	prof. Ben Van Calster	Statistics
	prof. Guy Bosmans	Clinical Psycology
	prof. Pascal Borry	Ethics
	prof. dr. Anne Smits	Paediatrics
	prof. dr. Anne Uyttebroeck	Paediatrics
	prof. dr. Ariel Alonso	Statistics (alternate)
	prof. dr. Benoit Nemery	Pneumology
	prof. dr. Gregor Verhoef	Haematology
	prof. dr. Jan Verhaegen	Laboratory Medicine
	prof. dr. Jan de Hoon	Clinical Pharmacology
	prof. dr. Karin Sipido	Experimental Cardiology
	prof. dr. Maria Schetz	Intensive care
	prof. dr. Simon Brumagne	Physiotherapy
	prof. dr. Xavier Bossuyt	Immunology
