# Supplementary material for: The effect of automated audit and feedback on data completeness in the electronic health record of the general physician: protocol for a cluster randomized controlled trial
Source: Trials. 2021 May 4;22:325. doi: 10.1186/s13063-021-05259-9 (PMC8097814; doi:10.1186/s13063-021-05259-9)
Supplement: Supplementary file 3 — Additional file 3. Informed consent (Dutch). [file 13063_2021_5259_MOESM3_ESM.docx]

**Audit en feedback voor huisartsen in het Intego netwerk - Informed consent document**

U wordt uitgenodigd om deel te nemen aan een studie binnen het Intego netwerk. Hieronder lichten wij de studie toe, vragen wij de toestemming voor uw deelname en delen wij ook onze contactgegevens mee. Voordat u akkoord gaat om aan deze studie deel te nemen, vragen wij u om kennis te nemen van wat deze studie zal inhouden op het gebied van organisatie, zodat u een welbewuste beslissing kunt nemen.

**Noodzakelijke informatie voor uw beslissing om deel te nemen**

Situering van het onderzoek

Binnen Intego, het project van ACHG – KU Leuven waaraan u reeds deelneemt, voorzien we na de dataophaling en datacleaning feedback over het registreren van parameters, diagnoses en handelingen in de elektronisch medische dossiers (EMD). Deze feedback heeft als doel het registratiegedrag van de huisarts te verbeteren/optimaliseren. Hierdoor trachten we de huisarts te stimuleren om de informatie in de EMD’s zo volledig en gestructureerd mogelijk te onderhouden door zo goed mogelijk gebruik te maken van codering in ICPC-2 en ICD-10 codering. Zo verkrijgt de huisarts een optimaal instrument om zicht te hebben op zijn/haar patiënten, vlotter te communiceren met collega’s binnen de huisartspraktijk en kan dit de transmurale communicatie verbeteren. Een bijkomend argument voor betere registratie en codering is dat een gestructureerd EMD ervoor zorgt dat de onderzoekers een beter beeld hebben op de patiënten uit de eerste lijn.

De feedback om zo’n gestructureerd EMD te bekomen, kan verschillende vormen aannemen. Audit en feedback is een uitgebreid onderzochte kwaliteitsinterventie, die volgens de laatste Cochrane-review leidt tot kleine maar potentieel belangrijke verbeteringen in de professionele praktijk. Er zijn aanwijzingen dat feedback de EMD-registratie kan verbeteren, maar het effect en de belangrijke kenmerken van feedback zijn nog steeds onderwerp van debat. Voorgaand werk heeft enkele hypotheses geïdentificeerd voor het ontwerpen van een audit en feedbackinterventie en suggesties voor het verbeteren van de effectiviteit van de interventie zijn beschikbaar in de literatuur. Er zijn verschillende criteria waaraan feedback zou kunnen voldoen om invloed te hebben op het registratieniveau van huisartsen in het EMD. We willen nu evalueren of de inspanning om een ​​uitgebreidere feedbackinterventie uit te voeren effect heeft op het registratiegedrag van de huisarts.

Onze onderzoeksvraag is: verbetert een audit en uitgebreide feedbackinterventie de kwaliteit van registratie in het EMD van de huisarts in vergelijking met standaardfeedback?

Concreet voor u als huisarts/huisartsenpraktijk

Binnen Intego voorzien we na de dataophaling en datacleaning feedback over het registreren van parameters, diagnoses, handelingen… Deze feedback kan verschillende vormen aannemen. Er wordt feedback voorzien voor alle huisartsen in de huisartspraktijken die in het Intego netwerk zitten. Deze huisartsen hebben een contract getekend om data op te halen uit hun EMD’s. Voor dit onderzoek zal enkel de vorm van feedback veranderd zijn. U krijgt te allen tijde nog feedback over het registreren in het EMD, alleen de vorm verandert.

Wij willen met onze studie het verschil in (een eventuele) verbetering in registratie voor de verschillende vormen van feedback nagaan. Zo kunnen we nagaan welke vorm van feedback we in de toekomst dienen te gebruiken.

Vertrouwelijkheid van de gegevens

Deze studie kadert binnen de dataophaling van het basis Intego project. Alle voorwaarden rond pseudonimisering van de data uit het contract met Intego zijn van toepassing.

Zoals alle medische gegevens worden ook de gegevens die in het kader van de studie verzameld worden uiterst vertrouwelijk behandeld. Uw gegevens zullen worden verwerkt overeenkomstig de Europese Algemene Verordening inzake Gegevensbescherming (AVG) en de Belgische Wetgeving betreffende de bescherming van natuurlijke personen met betrekking tot de verwerking van persoonsgegevens. Tine De Burghgraeve is de verwerkingsverantwoordelijke voor uw gegevens.

Intrekking van uw toestemming

U neemt vrijwillig deel aan deze studie en u hebt het recht om uw toestemming voor gelijk welke reden in te trekken. U hoeft hiervoor geen reden op te geven.

Als u uw toestemming intrekt, zullen de gegevens bewaard blijven die tot op het ogenblik van uw stopzetting werden verzameld. Dit om de geldigheid van de studie te garanderen. Er zal geen enkel nieuw gegeven aan de opdrachtgever worden gegeven.

Vragen / klachten / contact

Indien u vragen hebt over hoe wij uw gegevens gebruiken of uw recht op inzage, correctie, eventueel stopzetting van de verdere verwerking wil uitoefenen, dan kan u hiervoor steeds terecht bij uw arts-onderzoeker op volgend contactadres: Opdrachtgever van de studie: Academisch Centrum Huisartsgeneeskunde – KU Leuven

Contactpersoon: Tine De Burghgraeve, PhD

Academisch Centrum voor Huisartsgeneeskunde - KU Leuven
Kapucijnenvoer 33 blok j 3000 Leuven
Tel: +32 16 37 72 76
[tine.deburghgraeve@kuleuven.be](mailto:tine.deburghgraeve@kuleuven.be)

Indien u naderhand nog bijzondere aandachtspunten heeft of klacht wenst neer te leggen, kan u terecht bij het privacyteam van de KU Leuven op privacy@kuleuven.be.

Codering van de studiegegevens:

De verzamelde gegevens zullen geen elementen bevatten waarvan de combinatie tot de identificatie van de voorschrijvende huisarts/groepspraktijk zou kunnen leiden.

Goedkeuring door centrale en lokale commissies:

Deze studie werd goedgekeurd door een onafhankelijke ethische commissie (Ethische Commissie Onderzoek UZ/KU Leuven, die als centrale commissie fungeert voor dit project). Deze studie wordt uitgevoerd volgens de richtlijnen voor de goede klinische praktijk (ICH/GCP) en volgens de meest recente versie van de verklaring van Helsinki opgesteld ter bescherming van mensen deelnemend aan klinische studies. In geen geval dient u de goedkeuring door de Ethische Commissie Onderzoek UZ/KU Leuven te beschouwen als een aansporing tot deelname aan deze studie.

**Toestemmingsverklaring**

Ik, ondergetekende,

Naam/Voornaam..........................................................................Geboortedatum........................

Handelend in naam van huisartsenpraktijk ……………………………………………………………………………….

Adres praktijk: Straat ......................................................................................Nummer ................

Postcode ..............................Gemeente...................................................................................

geef, in mijn hoedanigheid van huisarts, de toestemming aan ACHG – KU Leuven om de gegevens uit de EMD’s van mijn praktijk te verwerken voor de Intego feedback studie.

Opgemaakt in ………. originelen, te ....................................op datum van ..…/……./...............

Ondergetekende bevestigt hiervan één origineel exemplaar ontvangen te hebben.

............................................................... [**voornaam + naam + handtekening ondergetekende**]
